# Long-term Trends in Mental Health Disorders After the 2011 Great East Japan Earthquake and Tsunami

**DOI:** 10.1001/jamanetworkopen.2020.13437

**Published:** 2020-08-14

**Authors:** Shiho Kino, Jun Aida, Katsunori Kondo, Ichiro Kawachi

**Affiliations:** 1 Harvard T.H. Chan School of Public Health, Department of Social and Behavioral Sciences, Boston, Massachusetts; 2 Tohoku University Graduate School of Dentistry, Department of International and Community Oral Health, Sendai, Japan; 3 Chiba University, Center for Preventive Medical Sciences, Chiba, Japan; 4 Department of Gerontological Evaluation, Center for Gerontology and Social Science, National Center for Geriatrics and Gerontology, Aichi, Japan

## Abstract

**Importance:**

Posttraumatic stress symptoms (PTSS) and depressive symptoms are common among survivors of a major disaster, yet few longitudinal studies have documented their long-term persistence at the community level.

**Objective:**

To examine the trajectories of PTSS and depressive symptoms for as long as 5.5 years after the 2011 Great East Japan earthquake and tsunami.

**Design, Setting, and Participants:**

This cohort study used data from 2781 participants in 3 waves of the Iwanuma Study (2010, 2013, and 2016), a cohort of community-dwelling older adults (≥65 years in August 2010) in Iwanuma, Japan, who were directly affected by the 2011 disaster. The baseline assessment of mental health predated the disaster by 7 months, and survivors were followed up for 5.5 years after the disaster. Data analysis was performed from October 2019 to February 2020.

**Exposures:**

The March 11, 2011, Great East Japan earthquake and tsunami.

**Main Outcomes and Measures:**

PTSS were measured by 9 questions on the Screening Questionnaire for Disaster Mental Health, while depressive symptoms were measured by 15 items on the Geriatric Depression Scale short form.

**Results:**

The analytic samples for trajectories of PTSS and depressive symptoms included 2275 and 1735 respondents, respectively. In the study population at baseline, there were slightly more women (1262 of 2275 [55.5%] and 882 of 1735 [50.8%]), and most participants were aged 65 to 74 years (1533 [67.4%] and 1224 [70.5%]) and married (1664 [76.2%] and 1319 [77.7%]). Overall, there was a 13.6% (95% CI, 11.7%-15.6%) incidence of depression among individuals who did not have depression before the disaster, while a further 11.1% (95% CI, 9.8%-12.4%) reported PTSS after the disaster. By 5.5 years of follow-up, approximately half of the survivors with new depressive symptoms in 2013 (85 of 168 [50.6%]) and PTSS (147 of 253 [58.1%]) had recovered. The prevalence of depression in the community remained remarkably stable (between 504 participants [29.0%] and 506 participants [29.2%]) comparing predisaster and postdisaster data.

**Conclusions and Relevance:**

In this study, mental illness symptoms persisted for more than 5 years among half of disaster survivors, but the community-wide prevalence of depression remained stable, suggesting that the community itself was resilient.

## Key Points

**Question:**

What is the persistence of mental illness symptoms among individuals affected by the 2011 Great East Japan earthquake and tsunami 5.5 years after the disaster?

**Findings:**

In a cohort study of 2781 community-dwelling older adults, approximately half of the individuals who reported posttraumatic stress symptoms and depression after the disaster had recovered by 5.5 years. The overall prevalence of depression remained stable between predisaster and postdisaster data.

**Meaning:**

In this study, although mental illness symptoms persisted for more than 5 years among half of disaster survivors, the community-wide prevalence of depression remained unchanged, suggesting that the community itself was resilient.

## Teaser

This cohort study examines the long-term trajectories of posttraumatic stress and depressive symptoms among older, community-living adults who were affected by the 2011 Great East Japan earthquake and tsunami.

## Introduction

It is well established that disasters are associated with an increase in many psychological problems, including depressive symptoms and posttraumatic stress symptoms (PTSS).^1^ However, the long-term prognosis of mental disorders following disaster remains sparsely documented. Few studies have followed survivors for more than 3 years.^2,3^ Even fewer studies have managed to capture the mental health status of survivors predating their disaster experiences,^2,3^ so we cannot determine whether the disaster caused worsening of mental health or whether poor mental health already predated disaster exposure in affected populations.

Absent a predisaster assessment of mental health status, accurate estimates of the incidence of mental illness are difficult to obtain.^4^ Asking survivors to retrospectively recall their mental health prior to disaster is obviously hampered by recall bias. In 1 of the few existing studies to document predisaster mental health, Fergusson et al^5^ followed survivors of the Canterbury earthquakes in New Zealand during 2010 and 2011. The researchers used the Christchurch Health and Development Study, a 35-year longitudinal birth cohort (with 635 men and 630 women), in which predisaster mental health information was available among survivors. The study found that the prevalence of some types of mental disorders (ie, major depression, posttraumatic stress disorder, other anxiety disorders, and nicotine dependence) was significantly elevated among people who experienced traumatic events during the earthquakes. However, after adjusting for confounders that were assessed prior to the earthquakes, the excess risk of mental disorders stemming from disaster-related experiences was substantially attenuated, with the exception of nicotine dependence.

Turning to the issue of long-term prognosis of mental illness in the aftermath of a disaster, Morina et al^3^ conducted a systematic review of remission rates from posttraumatic stress disorder in adults. Among 42 studies with a mean follow-up period of 40 months, they found that posttraumatic stress disorder following disaster-related trauma had the highest mean remission rate (60.0%) compared with remission of symptoms related to physical disease (31.4%).^3^ More specifically, 5 natural disaster–related studies were found, with an mean period between the disaster event and follow-up assessment of 21 months (the range for the period between trauma and baseline assessment was 1.5-6.5 months, and the range for the period between baseline assessment and follow-up was 12-30 months.).^3^ However, none of those studies assessed mental health status prior to the disaster.^6,7,8,9,10^

According to the findings from a seminal review by Norris et al,^11^ psychological symptoms peak in the first year after the disaster and tend to improve as time passes, while a delayed onset of the disorder is less common. Twelve years after Hurricane Katrina, Raker et al^12^ found that 39% and 29% of people had recovered from PTSS and depressive symptoms, respectively, while 3% and 9% experienced delayed onset of PTSS and incident depressive symptoms. On the other hand, 14% and 28% had persistent PTSS and depressive symptoms even 12 years after the disaster.^12^

The factors associated with persistence vs remission of mental illness symptoms remain poorly understood, especially with regard to risk and protective factors that predate the disaster experience. Iwanuma city in Miyagi prefecture was 1 of the localities directly affected by the tsunami on March 11, 2011. The Iwanuma Study is a community-based longitudinal cohort established in 2010 that has collected information every 3 years. A unique feature of the Iwanuma Study is that baseline information from participants was assessed 7 months before the disaster and the follow-up rate among survivors remains very high. In a series of previous reports,^13,14,15,16^ we documented that levels of social capital predating the disaster were associated with a lower incidence of PTSS and depressive symptoms. However, our previous reports involved relatively short observation periods (approximately 2.5 years postdisaster) while most of the survivors were still housed in temporary accommodation. In the present study, we sought to examine long-term prognosis of PTSS and depressive symptoms among older individuals who experienced the 2011 disaster.

## Methods

### Study Population

The Iwanuma Study is a community-based longitudinal cohort established in 2010, when the city of Iwanuma in Miyagi prefecture (population 44 187 in 2010) was selected as 1 of 31 field sites for a nationwide cohort study of healthy aging in Japan, called the Japan Gerontological Evaluation Study (JAGES).^17^ The baseline of the Iwanuma Study was collected in August 1 to 20, 2010, when a census was undertaken of all the city’s residents aged 65 years or older. The names and addresses of residents were obtained from municipal records, and the baseline postal survey inquired about sociodemographic information, health habits, health status, and people’s social connections to others. The response rate to the baseline survey was 59.0% (5058 of 8576 residents), which is comparable to similar surveys of community-dwelling residents. Comparison of the baseline characteristics of our sample with the general population of the city (assessed from the local government census) confirmed that the Iwanuma Study participants were representative with respect to sex and age distribution as well as employment status.^14^

**Figure 1.**
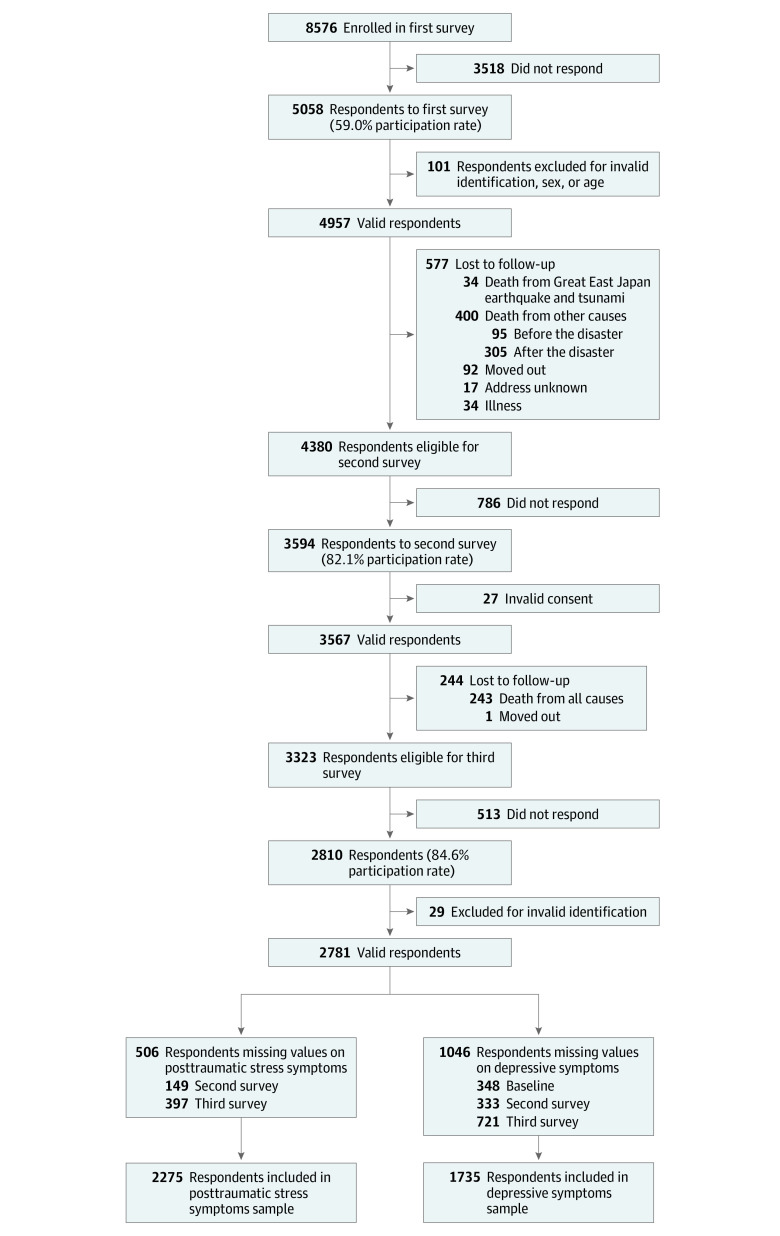
Study Flowchart Summing the individuals with missing values does not match the total number because some individuals had missing values on more than 1 covariate.

As summarized in the flowchart (Figure 1), a total of 4957 individuals responded to the baseline (predisaster) survey, 3567 individuals responded to the second (postdisaster) survey (October 1, 2013, to January 31, 2014), and 2810 individuals to the third survey (November 14, 2016, to December 5, 2016). We excluded people whose answers of sex and age were substantially different because the same people might not have answered.

A total of 34 original participants died on the day of the disaster. Among the 2781 valid responders to the 2016 survey, 506 participants (18.2%) did not complete the PTSS questions on either second or third survey, while another 1046 participants (37.6%) did not complete depressive symptoms either at baseline, second survey, or third survey. This left 2275 and 1735 individuals for the analyses of trajectories of PTSS and depressive symptoms, respectively, across 3 survey waves.

The research protocol was reviewed and approved by the human participants committees of the Harvard T.H. Chan School of Public Health, Tohoku University, Nihon Fukushi University, and Chiba University. Participants provided written informed consent. All data were deidentified. This study followed the Strengthening the Reporting of Observational Studies in Epidemiology (STROBE) reporting guideline for cohort studies.

### Outcomes

#### PTSS

PTSS was measured with the Screening Questionnaire for Disaster Mental Health, which was originally developed to screen for posttraumatic stress disorder and depression and subsequently validated for older populations in the 1995 Great Hanshin Earthquake in Japan.^18^ PTSS was assessed with 9 questions with binary answers (ie, yes/no) (Box). Affirmative responses were summed (range, 0-9), and the guideline suggests that a score of 6 or more is considered severely affected by the event and possibly with posttraumatic stress disorder, with an area under the receiver operating curve of 0.91 (95% CI, 0.83, 0.99) compared with the criterion-standard Clinician Administered Posttraumatic Stress Disorder Scale. Thus, the reliability and validity of this measure are reasonably high, and this measure was used in our previous studies.^14,16^

Box. **Questions to Measure Posttraumatic Stress Symptoms (PTSS) and Depressive Symptoms**

**The Screening Questionnaire for Disaster Mental Health–9 items for PTSS**

Do you have trouble falling asleep or sleeping through the night?
Do you have nightmares about the event?
Do you feel irritable?
Do you feel that you are hypersensitive to small noises or tremors?
Do you avoid places, people, topics related to the event?
Do you think about the event when you do not want to?
Do you have trouble enjoying things you used to enjoy?
Do you get upset when something reminds you of the event?
Do you notice that you are making an effort to try not to think about the event, or are trying to forget it?

**The Geriatric Depression Scale Short Form–15 items**

Are you basically satisfied with your life?^a^
Have you dropped many of your activities and interests?
Do you feel that your life is empty?
Do you often get bored?
Are you in good spirits most of the time?^a^
Are you afraid that something bad is going to happen to you?
Do you feel happy most of the time?^a^
Do you often feel helpless?
Do you prefer to stay at home, rather than going out and doing new things?
Do you feel you have more problems with memory than most?
Do you think it is wonderful to be alive now?^a^
Do you feel pretty worthless the way you are now?
Do you feel full of energy?^a^
Do you feel that your situation is hopeless?
Do you think that most people are better off than you are?

^a^These questions were reverse coded, ie, a lower score indicated more severe depressive symptoms.

#### Depressive Symptoms

Depressive symptoms were measured by the Geriatric Depression Scale Short Form, which was developed to measure depressive symptoms among older adults.^19,20^ The form has 15 items with binary answers (ie, yes/no) (Box). Responses were summed (range, 0-15) to assess depressive symptoms, with higher scores indicating more severe depressive symptoms. This scale was validated to screen major depressive symptoms (ie, a score of 5 or more) with an area under the receiver operating characteristic curve of 0.94 (sensitivity, 92%; specificity, 87%) against the Structured Clinical Interview for the *Diagnostic and Statistical Manual of Mental Disorders, Third Edition, Revised*, as the criterion standard.^21^ This measure has been validated to assess depressive symptoms among older Japanese populations.^13,14,15^

#### Trajectories of PTSS and Depressive Symptoms

Following Norris et al^11^ and Raker et al,^12^ we categorized mental health trajectories into 4 types: never experienced PTSS or depressive symptoms, recovered from PTSS or depressive symptoms, had delayed onset of PTSS or depressive symptoms, and had persistent PTSS or depressive symptoms. For PTSS, the never group included individuals who did not report PTSS in either 2013 or 2016, while the delayed onset group included individuals who did not report PTSS in 2013 but had developed symptoms in 2016. The recovered group included those with PTSS in 2013 but not in 2016. Finally, the persistent group included those reporting PTSS in both 2013 and 2016.

Questions regarding depressive symptoms were asked in all 3 waves; therefore, following the methods of Raker et al,^12^ we categorized the possible trajectories as follows: the never group included individuals without depressive symptoms in any of the 3 waves (2010, 2013, and 2016). The delayed onset group included people who did not report depressive symptoms in 2010 or 2013 but developed symptoms by 2016. The recovered group consisted of individuals who reported significant depressive symptoms (ie, Geriatric Depression Scale score of ≥5) in 2010 and/or 2013 but did not report them (ie, Geriatric Depression Scale score of ≤4) in 2016, ie, their symptoms had remitted. The persistent group included those reporting depressive symptoms at least once in 2010 or 2013 as well as in 2016, meaning that this group included those with depressive symptoms in 2010 and 2016, in 2013 and 2016, and in 2010, 2013, and 2016.

### Statistical Analysis

First, we examined the overall community prevalence of mental illness symptoms at each survey. Then we described the within-individual trajectories of PTSS across the 2 waves and depressive symptoms across the 3 waves. We conducted paired *t* tests to examine the statistical significance in the prevalence of mental illness symptoms.

We imputed the missing values of covariates through the Markov chain Monte Carlo methods using mi command in Stata. We created 20 imputed data sets and used the mi estimate command to estimate model parameters from multiply imputed data. All the analyses were performed using Stata version 15 (StataCorp) from October 2019 to February 2020. Statistical significance was set at *P* < .05, and all tests were 2-tailed.

## Results

The analytic samples for trajectories of PTSS (from 2013-2016) and depressive symptoms (from 2010-2016) included 2275 and 1735 respondents, respectively (Figure 1). The Table exhibits the characteristics of the samples for each analysis. At baseline, there were slightly more women (1262 of 2275 [55.5%] and 882 of 1735 [50.8%]), and most participants were aged 65 to 74 years (1533 [67.4%] and 1224 [70.5%]) and married (1664 [76.2%] and 1319 [77.7%]). Nearly 70% had more than 9 years of schooling (1550 [68.8%]) and 1205 [70.0%]), and most had equivalized household income of ¥200 million or more (≥$1 869 050) (1381 [52.8%] and 1054 [54.7%]).

**Table.** Demographic Characteristics of Analytic Sample

Characteristic	Participants, No (%)	
	For PTSS trajectory (n = 2275)	For DEP trajectory (n = 1735)
Sex		
Men	1013 (44.5)	853 (49.2)
Women	1262 (55.5)	882 (50.8)
Baseline age, y		
65-74	1533 (67.4)	1224 (70.5)
≥75	742 (32.3)	511 (29.5)
Education, y		
≤9	710 (31.2)	514 (30.0)
>9	1550 (68.8)	1205 (70.0)
Baseline equivalized income, millions of ¥		
<200	894 (47.2)	681 (45.3)
≥200	1381 (52.8)	1054 (54.7)
Baseline marital status		
Single	542 (23.8)	387 (22.3)
Married	1664 (76.2)	1319 (77.7)
Trajectory group		
Never	1913 (84.1)	962 (55.4)
Delayed	109 (4.8)	101 (5.8)
Recovered	147 (6.5)	268 (15.4)
Persistent	106 (4.7)	404 (23.3)

Abbreviations: DEP, depressive symptoms; PTSS, posttraumatic stress symptoms.

### Trends in Community Prevalence of Mental Illness

**Figure 2.**
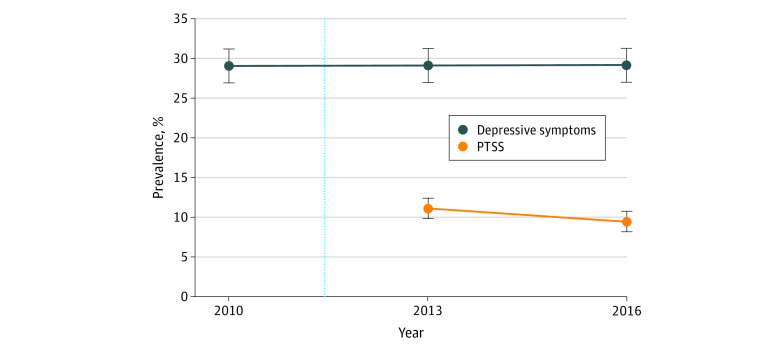
Prevalence of Posttraumatic Stress Symptoms (PTSS) and Depressive Symptoms Error bars indicate 95% CIs.

Figure 2 presents the trends in community prevalence of PTSS and depressive symptoms in Iwanuma from 2010 (before the disaster) to 2016 (5 years after the disaster). The prevalence of people with depressive symptoms was remarkably stable between 2010 and 2016 (approximately 29%), which was statistically confirmed (2010 vs 2013, 504 [29.0%] vs 506 [29.2%]; *P* = .91; 2013 vs 2016, 506 [29.2%] vs 505 [29.1%]; *P* = .96). The prevalence of PTSS declined from 2013 to 2016, from 253 (11.1%) to 215 (9.5%) (*P* = .02). Combining the prevalence of depressive symptoms and PTSS, approximately 40% of survivors were experiencing significant mental health symptoms (PTSS or depressive symptoms) more than 5 years after the disaster. However, this observation is tempered by the fact that the prevalence of depressive symptoms was already quite high before the disaster (504 [29.0%]), so we cannot attribute most of the excess mental illness symptoms to the disaster.

### Within-Individual Trajectories of Mental Illness Symptoms

**Figure 3.**
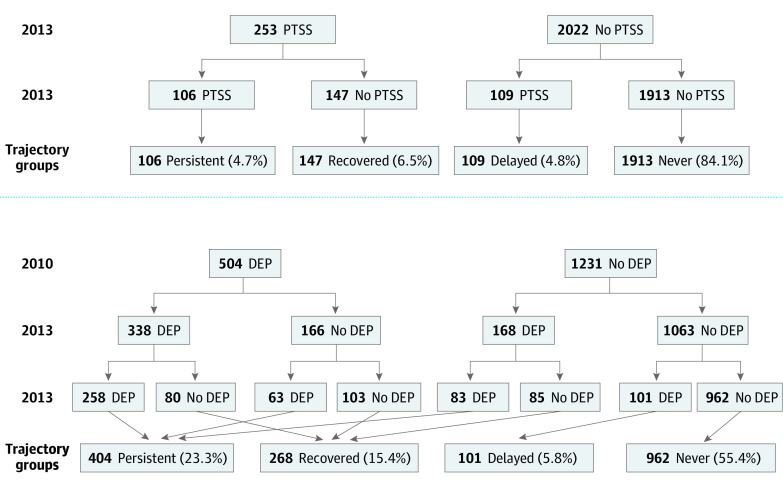
Trajectories of Posttraumatic Stress Symptoms (PTSS) Among 2275 Participants and Depressive Symptoms (DEP) Among 1735 Participants

Figure 3 summarizes the within-individual trajectories of PTSS and depressive symptoms between 2010 and 2016. Overall, there was a 13.6% (95% CI, 11.7%-15.6%) incidence of depression among people who did not have depression before the disaster (n = 168). However, among 504 people who reported significant depressive symptoms before the disaster, 166 (32.9%) experienced symptom remission by 2013. Between 2013 and 2016, a further 165 people remitted, while 164 people experienced delayed onset of depression. Among those with preexisting depressive symptoms at baseline in 2010, 338 (67.1%) had persistent depressive symptoms in 2013, and more than half had persistent depressive symptoms in 2013 and 2016 (258 [51.2%]). By 2016, 85 of 168 participants (50.6%) who first reported depressive symptoms in 2013 had recovered.

A total of 253 survivors (11.1%; 95% CI, 9.8%-12.4%) reported serious PTSS in 2013, of whom 106 (41.9%) still had PTSS in 2016, while 147 (58.1%) had recovered by 2016. Of 2022 with no PTSS in 2013, 109 (5.3%) had developed symptoms by 2016. We compared the prevalence of PTSS in 2013 (11.1%) and in 2016 (9.5%) and found that null hypothesis was rejected with a *P* = .02 from a 2-tailed paired *t* test. Therefore, we concluded that the prevalence of PTSS in 2013 was statistically associated with its prevalence in 2016. Overall, 1913 participants (84.1%) did not have PTSS either in 2013 or 2016. We also conducted a sensitivity analysis to examine the prevalence of PTSS only among those with information on depressive symptoms. We found that 163 out of 1686 (9.7%) in 2013 and 127 of 1583 (8.0%) in 2016 had PTSS, indicating a similar trend as our main findings. In addition, to consider selection bias owing to missing data, we conducted inverse-probability weighting as a sensitivity analysis. We found that the prevalence of depressive symptoms was 29.5% (95% CI, 27.3%-31.7%) in 2010, 29.6% (95% CI, 27.4%-31.8%) in 2013, and 29.2% (95% CI, 27.0%-31.4%) in 2016 and the prevalence of PTSS was 11.1% (95% CI, 9.7%-12.4%) in 2013 and 9.2% (95% CI, 8.0%-10.4%) in 2016. These findings are in line with our main findings.

## Discussion

The mental health of survivors of a traumatic event, such as a natural disaster, has been extensively studied.^22^ However, owing to the lack of predisaster information, very few studies have been able to tease out the consequences of disaster exposure from preexisting conditions. In this study, we examined the community-level prevalence of mental health disorders as well as the within-individual trajectories of depressive symptoms and PTSS following the 2011 Great East Japan earthquake and tsunami. We have previously reported^13,14^ that personal exposure to trauma (loss of relatives, housing damage) was associated with new onset depressive symptoms and PTSS. In contrast to these individual experiences, from a community-wide perspective, we found little evidence in the present study of an epidemic of depressive symptoms after the disaster.

There are 3 possible reasons for the apparent stability in the prevalence of depression between predisaster to postdisaster surveys. First, the prevalence of depressive symptoms was already high in this community before the disaster (29%). Although this figure seems high, our data are consistent with previous studies among older Japanese populations using the same instrument and the same cutoff point (eg, Wada et al^23^ reported 33.5% and Sasaki et al^24^ reported between 21.5% and 36.2%). Interestingly, in the absence of predisaster information, researchers might have been tempted to conclude that the high prevalence of depression in the community was attributable to the traumatic experiences associated with the disaster. The second reason why the community prevalence of depressive symptoms appeared to be stable over time is because even though 13.6% of residents experienced new onset of depression after the disaster, this was offset by individuals who were depressed before the disaster but whose symptoms had remitted by follow-up. A third possible explanation for the stability of the prevalence of depression may be due to posttraumatic growth that some individuals experience after a disaster.^25^ For example, some reports suggest that people may become more socially active following disaster experiences and that increased social capital might promote improved mental resilience.^26^

There are 2 caveats to this interpretation. First, we did not observe the immediate consequences of the disaster in the very short term (eg, within the first year), during which people were more likely to suffer from acute grief and distress given the fact that the first year after a disaster is the peak of psychological symptoms.^11^ Second, there may have been some selective attrition of individuals who were depressed before the disaster. In a previous report, we found that individuals who were severely depressed on the day of the earthquake and tsunami were twice as likely to lose their life, possibly as a result of delayed evacuation.^27^ The selective mortality of these individuals would tend to underestimate the true counterfactual prevalence of depression in the community after the disaster. On the other hand, the consequence of this bias is likely to be small, given that a total of 34 of the baseline sample of 4857 participants died (yielding a 0.69% mortality rate on the day of the disaster).

Regarding PTSS, we found that approximately 60% of people who reported PTSS in 2013 had recovered by 2016. This is consistent with the findings of the meta-analysis among natural disaster survivors by Morina et al.^3^

When the figures for depressive symptoms and PTSS are combined, our data suggest that nearly 40% of survivors were still experiencing significant mental health symptoms (PTSS or depressive symptoms) more than 5 years after the disaster. While this figure presents a scenario of persistent, long-term mental health problems in the wake of the disaster, much of it cannot be attributed to the disaster trauma given that nearly one-third of the sample reported significant depressive symptoms preceding the March 11, 2011, event. In other words, we did not find evidence of a community-wide epidemic of mental illness following a major disaster.

### Strengths and Limitations

The major strength of this study was that we collected the baseline mental health data before the disaster. As Galea et al^4^ have argued, study designs featuring only postdisaster assessments are unable to distinguish between mental health consequences of disaster vs the persistence of predisaster conditions. Our 3-wave analysis also allowed us to examine the within-individual trajectories of mental illness symptoms during a longer period than in most previous studies.

There are some limitations of our study. First, the response rate at the baseline survey was 59.0%, which is quite similar compared with other community-dwelling surveys. Furthermore, the characteristics of the baseline sample were very similar to 2010 National Census population in the area.^14^ The response rates at follow-up surveys were very high (82.1% and 84.6% for the 2 follow-up survey waves, respectively). Second, there might be a chance of selection bias due to loss to follow-up during 3 years. People who were most depressed or had severe PTSS were more likely to have dropped out, which could potentially inflate the recovery rates. However, our sensitivity analysis showed similar results as our main findings.

## Conclusions

In this study, approximately 40% of survivors from the Great East Japan earthquake and tsunami still experienced PTSS or depressive symptoms or both 5.5 years after the disaster. However, the mental resilience at the community level was remarkably stable, suggesting that the community itself was resilient.
